# Correlation between the Crosslink Characteristics and Mechanical Properties of Natural Rubber Compound via Accelerators and Reinforcement

**DOI:** 10.3390/polym12092020

**Published:** 2020-09-04

**Authors:** Do Young Kim, Jae Woo Park, Dong Yun Lee, Kwan Ho Seo

**Affiliations:** Department of Polymer Science and Engineering, Kyungpook National University, Daegu 41566, Korea; ddyykk9655@gmail.com (D.Y.K.); yellon3375@gmail.com (J.W.P.)

**Keywords:** natural rubber, crosslink characteristics, mechanical properties, thermal aging, accelerator, reinforcement agent

## Abstract

The extreme elasticity and reversible deformability of rubber, which is one of the most versatile polymers in modern society, is dependent on several factors, including the processing conditions, curing system, and types of additives used. Since the rubber’s mechanical properties are influenced by the existing structural crosslinks, their correlation with the crosslink characteristics of rubber was investigated using the equilibrium swelling theory of the Flory–Rehner equation and the rubber–filler interaction theory of the Kraus equation. Herein, we examined whether the accelerator and reinforcement agent quantitatively contributed to chemical cross-linkages and rubber–filler interaction. In conclusion, the accelerator content supported the chemically crosslinked structures of the monosulfides and the disulfides in natural rubber (NR). Additionally, these results demonstrated that the mechanical properties and the thermal resistance of NR were dependent on the crosslink characteristics. The findings of this study provide an insight into the development and application of NR products for the mechanical optimization of rubber-based products.

## 1. Introduction

Natural rubber (NR), which is obtained from *Hevea brasiliensis* as latex in nature, is one of the oldest polymers in use and consists mainly of a cis-1,4-polyisoprene structure [1]. Before crosslinking reactions, NR exhibits low mechanical and thermal hardiness, is soft at high temperatures, and brittle at low temperatures. Conversely, crosslinked NR is renowned for its elasticity, high reversible deformability, good mechanical strength, and thermal hardiness [2,3,4] as crosslinking prevents the rubber chains from sliding and becoming entangled in each other under load [5,6]. The elasticity of NR makes it ideal for numerous applications in the production of tires, hoses, automobile parts, and household items [5]. In other words, the mechanical properties of rubber are directly related to the crosslink density and the overall structure of the rubber.

Crosslinked rubber generally consists of linkages brought about by sulfur-based chemical reactions between the rubber’s main chains, and are composed of monosulfide (–S–), disulfide (–S–S–), and polysulfide (–S_x_–, x ≥ 3). Since the mechanical properties of rubber are dependent on the structure and density of the crosslinks within the rubber [7], the properties of the material can be controlled via the use of additives such as fillers and accelerators, which can vary depending on the intended use. Relative to other reinforcement agents such as silica, clay, and inorganic additives, carbon black is widely used in the rubber industry as it is the most effective and inexpensive way to improve the rubber’s physical properties [8,9]. Accelerators are classified as primary or secondary. Primary accelerators, such as thiazole derivatives and sulfenamides, generate radicals within the rubber, thereby providing a reaction site for the crosslinking agent. Secondary accelerators, such as guanidine and triuram, boost the speed and state of the crosslinking reactions by activating the primary accelerator [10,11,12].

The equilibrium swelling theory [13] and thiol–amine analysis [7] are integral for conducting a chemical analysis of the structural crosslinks of NR. The thiol–amine reaction induces selective chain cleavage of the polysulfide and disulfide crosslinks. The equilibrium swelling theory, which accounts for entropy changes due to polymer–solvent mixing, is used to estimate crosslinks in polymers immersed in particular solvents. These methods require different solvents and materials, depending on the rubber sample and crosslinking agent being investigated. Other methods can be employed for conducting structural analyses of the crosslinks in polymers by using the Mooney–Rivlin model [14,15,16,17] and nuclear magnetic resonance [18,19].

In this study, the crosslink density and structure of NR were classified and quantified in relation to the addition of accelerators and reinforcement agents. This was done to accurately determine the effects exerted on the mechanical properties and thermal stability of NR, and to establish any correlation between the additives and the properties of NR. Herein, NR compounds were prepared with various amounts of carbon black and various accelerators. The crosslink density, structure, and mechanical properties of the resulting NR samples were characterized using the equilibrium swelling theory and thiol–amine analyses. The effects of these additives on the crosslink characteristics and mechanical properties of NR were also investigated.

## 2. Materials and Methods

### 2.1. Materials

NR was obtained from Karthik Rubbers (Ribbed smoked sheets 3, Bantwal, India). Carbon black (FEF grade: N550, surface area: 79 m^2^/g, density: 1.86 g/cm^3^; OCI, Seoul, Korea), stearic acid (OCI, Korea), sulfur (MIDAS SP325; Miwon Chemicals, Anyang, Korea), N-isopropyl-N′-phenyl-phenylenediamine (Shandong Sunshine Chemical, Liaocheng, China), and 2,2,4-trimethyl-1,2-dihydroquinoline (Shandong Sunshine Chemical) were employed as the reinforcement agent, lubricant, crosslinking agent, and antioxidants, respectively. Commercial grade 2,2′-dithiobis(benzothiazole) (MBTS) and diphenylguanidine (DPG) were used as the accelerators. Tetrahydrofuran (THF) and *n*-hexane, which were used for sample purification, and toluene, which was used for sample swelling, were all obtained from Sigma-Aldrich (St. Louis, MO, USA). For the structural analysis of the crosslinks, 2-propanethiol (≥98%), 1-dodecanethiol (≥98%), and piperidine (99%) were provided by Sigma-Aldrich.

### 2.2. Preparation of the NR Compounds

The NR compounds were prepared in five steps. First, the NR was finely cut and masticated using a 3 L dispersion kneader (Kansai Roll Co., Osaka, Japan) for 1 min. Reinforcement agents were added, and the whole was mixed for 3 min, followed by the addition of the lubricant and subsequent mixing for another 3 min. Next, the crosslinking agent was added and mixed at a rotor speed of 30 rpm until the temperature reached 90 °C. In the final step, the compound was mixed with the crosslinking accelerators in an 8-inch open-type roll (Bong Shin Co., Incheon, Korea) for 4 min at 30 °C. The five-step process was designed to prevent early curing and to improve the dispersion of the additives. The formulations and mixing conditions are presented in Table 1 and Table 2.

### 2.3. Curing the NR Compounds

The optimum curing time (t_90_) was determined by investigating the curing characteristics of NR compounds using an RLR-3 moving-die rheometer (Toyoseiki, Osaka, Japan). This was done to determine the scorch time (t_s2_), minimum torque (M_L_), maximum torque (M_H_), and t_90_ of the compounds of interest. Here, the NR compounds were shaped into squares with a length of 150 mm and a thickness of 2 mm using a laboratory hydraulic press (Model 3851, Fred S. Carver Inc., Menomonee Falls, WI, USA), and heated at 180 °C under 2000 psi for t_90_. The crosslinking analysis was conducted on NR samples cut into squares with a length of 10 mm and a thickness of 2 mm.

### 2.4. Treatment of the NR Samples for Crosslink Analysis

Various organic additives and impurities in the NR samples were removed by alternately soaking in *n*-hexane for 3 days and THF for 2 days at room temperature. After the extraction process, the samples were dried for 2 days at room temperature in a vacuum oven before being weighed. The crosslink density analysis was conducted on the dried NR samples, which had become swollen after a 3-day toluene soak at room temperature. Afterward, the weights of the swollen samples were measured. The crosslink structures of the NR samples were investigated using thiol–amine reactions, which were used to conduct structural analysis through the selective cleavage of certain sulfides by thiyl radicals. Polysulfide cleavage was achieved by reacting the swollen NR samples with a solution composed of 2-propanethiol (0.4 M) and piperidine (0.4 M) in toluene for 2 h under N_2_ gas. Cleavage of the polysulfide and disulfide was done by reacting the swollen NR samples with a solution composed of 1-dodecanethiol (1.0 M) in piperidine for 24 h under N_2_ gas [20]. Once the thiol–amine reactions were complete, the samples were removed from their respective solutions and washed with toluene. The crosslink analyses were performed on swollen NR samples after immersion in toluene for 24 h. The weights of the respective samples were measured and calculated according to the same procedure stated above.

### 2.5. Analysis of the Crosslink Density and Structure

The equilibrium swelling theory (as defined by the Flory–Rehner equation) and the rubber–filler interaction theory (as described by the Kraus equation) were used to quantitatively determine the crosslink density and structure of the rubber samples, respectively. The crosslink density $\left(\nu_{cross}\right)$ was calculated using the Flory–Rehner Equation (1) [13]:

(1) $$\nu_{cross}\;\left(mol/g\right)=\frac{1}{2M_{c}}=-\frac{\ln\left(1-\nu_{r}\right)+\nu_{r}+\chi \nu_{r}^{2}}{2\rho_{r}V_{s}\left(\sqrt[{3}]{\nu_{r}}-\frac{\nu_{r}}{2}\right)}$$

where $M_{c}$ is the average molecular weight of the rubber between the crosslinks, $V_{r}$ is the volume fraction of the equilibrium swollen rubber, χ describes the Flory–Huggins polymer–solvent interaction parameter, $V_{s}$ is the molar volume of the solvent used (i.e., 106.27 cm^3^/mol for toluene), and $\rho_{r}$ (g/cm^3^) is the density of rubber. Additionally, $V_{r}$ and χ were determined from Equations (2) and (3) [21,22]:

(2) $$\nu_{r}=\frac{\frac{W_{before}-W_{filler}}{p_{r}}}{\frac{W_{before}-W_{filler}}{p_{r}}+\frac{W_{after}-W_{before}}{p_{s}}}$$

where $W_{before}$ (g) is the weight of the rubber sample before swelling, $W_{after}$ (g) is the weight of the rubber sample after swelling, $W_{filler}$ (g) is the weight of the filler, and $p_{s}$ (g/cm^3^) is the density of the solvent.

(3) $$\chi =\beta +\frac{V_{s}}{RT}{\left(\sigma_{p}-\sigma_{s}\right)}^{2}$$

where β is the lattice constant for the polymer–solvent blends (i.e., β=0.34), *R* is the gas constant, *T* (K) is the absolute temperature (293.15 K), $\sigma_{p}$ (MPa^1/2^) is the solubility parameter of the rubber sample (16.7 MPa^1/2^ for NR), and $\sigma_{s}$ (MPa^1/2^) is the solubility parameter of the solvent (18.0 MPa^1/2^ for toluene) [6,23]. The calculated value of χ was 0.414. The Kraus equation was used to distinguish between the chemical crosslinking density by the crosslinking agent and the rubber–filler interaction by the reinforcement agent [24,25].

(4) $$\frac{\nu_{r-f}}{\nu_{r}}=1-m\left(\frac{\varphi }{1-\varphi }\right)$$

Here, $\nu_{r-f}$ is the volume fraction of the equilibrium swollen rubber without the filler, $\nu_{r}$ is the volume fraction of the equilibrium swollen rubber with the filler, m is the Kraus interaction between the rubber and the filler, and φ is the volume fraction of the filler. m was calculated by conducting linear regression analysis on the amount of the filler.

### 2.6. Instrumentation and Equipment

The curing characteristics were determined using a moving-die rheometer. The change in torque at 180 °C was measured for 12 min. An electronic densimeter (MD-200S, Mirage, Osaka, Japan) was used to measure the density of the samples via the water displacement method, and the given density values presented in this study were the average of six independent measurements. The mechanical properties were measured according to American Society for Testing and Materials (ASTM) D412-98a using a universal testing machine (ST-1001, Salt Inc., Incheon, Korea). All tests were performed at a crosshead speed of 500 mm/min and room temperature. Dumbbell-shaped samples were prepared according to ASTM D2240 and D412, and the average values from three different measurements obtained under the same test conditions were used. The dumbbell-shaped samples were exposed to air at 100 °C for 70 hours in a geer-type oven (No. 272, Toyoseiki, Japan). The thermal aging properties of the samples were investigated by comparing their mechanical properties before and after thermal aging according to ASTM D573.

## 3. Results and Discussion

### 3.1. Curing Characteristics of NR Compounds

The curing characteristics of rubber play an important role in rubber processing and lay the groundwork for the basic analysis of rubber processing systems because the curing behavior is heavily dependent on the type of rubber and additives used, as well as the amount of additives. Additionally, the best-performing rubbers depend on their mechanical and thermal properties, which are determined by measuring the t_s2_, t_90_, M_L_, and M_H_ values associated with these compounds. The t_s2_ indicates that rubber compound can be mixed at a given temperature before curing begins, and is defined as 5% of the M_L_ at M_H_. The t_90_ is the vulcanization time required to facilitate the optimal characteristics of the final products, and is defined as 90% of the M_L_ at M_H_ [26,27,28]. The curing characteristics of NR compounds with varying carbon black content and crosslinking accelerators are given in Table 3. The NR compounds showed decreased t_s2_ and t_90_, and an increase in the content of the DPG and the MBTS was attributed to the preferential promotion of the curing reaction by DPG rather than by MBTS. Similarly, these values showed a slight decrease when the carbon black content was increased; this was attributed to accelerated crosslinking reactions between rubber and sulfur via the generation of radicals at low temperatures with an increase in the amount of the crosslinking accelerator [29]. Additionally, this can be related to the fact that the carbon black strongly interacted with NR on the surface area of the carbon black [20].

The torque values are indicative of the shear force resistance in rubber at a given temperature. Although the M_H_ increased at higher amounts of DPG and carbon black, MBTS seemed to exert very little influence. The M_L_ was significantly influenced by the carbon black content, but the DPG content seemed to be irrelevant. Further, the addition of MBTS reduced M_L_ because MBTS acted as a plasticizer before decomposition and during the vulcanization reaction [30]. The relative crosslink density of rubber can be determined by examining the torque difference between M_H_ and M_L_, and was indicative of the formation of crosslinks during the curing reaction [31,32,33]. This difference was increased by increasing the DPG, MBTS, and carbon black content. Consequently, both the accelerators and fillers affected the relative crosslink density. The rate of the curing reaction was primarily influenced by the accelerators and the filler, which affected the torque observed during the curing reaction.

### 3.2. Crosslink Characteristics of NR Compounds Calculated Using the Flory–Rehner Equation

**Figure 1:**
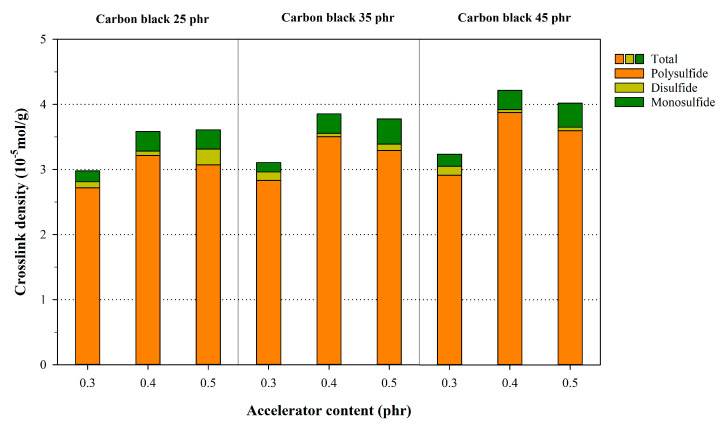
Variations of the crosslink structures in the NR sample with varying accelerator and carbon black content, as calculated using the Flory–Rehner equation.

As mentioned earlier, crosslink reactions are important in improving the overall performance of rubber. Analyzing the crosslink density and structure of rubber provides insight that can be used to enhance the polymer’s performance. In a rubber chain, sulfur reacts to form monosulfides, disulfides, and polysulfide structures, and the crosslink density is significantly affected by the type and amount of the fillers, accelerators, and activators used [29]. These factors contribute to variations in the crosslink structures and densities, resulting in various mechanical and thermal properties. Herein, the crosslink densities and structures of NR compounds with various accelerators and filler contents were evaluated using the Flory–Rehner equation. These variations in the crosslink densities and structures are shown in Figure 1. The total crosslink density was calculated as the sum of the polysulfide, disulfide, and monosulfide content. As it can be seen, when the amounts of the accelerator and carbon black were increased, the total crosslink density of the polymer increased. In particular, the total crosslink density increased sharply when the content of carbon black in the polymer rose from 25 to 35 per hundred resin (phr). This could be explained by the rubber–filler interaction [34,35] and the equilibrium swelling theory. Here, the immersion of the crosslinked rubber in a solvent enables the solvent molecules to penetrate the rubber chain. Crosslinked rubber with a high crosslink density can accommodate a lower number of solvent molecules by the immobilization of rubber chains, resulting in relatively less swelling. The addition of more carbon black increased the rubber–filler interaction, and the NR exhibited less swelling as a result. Additionally, the crosslink density tended to increase upon increasing the content of the accelerator. The number of polysulfide in the crosslink structure increased when the content of the accelerator and carbon black rose. From these results, it was attributed that a part of the change in the crosslink density were factors of the carbon black content, and was attributed to the swelling ratio caused by the rubber–filler interaction. This differed from the chemical crosslink bonds. Therefore, it was important to apply the Kraus equation to the Flory–Rehner equation to accurately conduct the chemical crosslink density and structure analysis and determine the influence of the accelerator content.

### 3.3. Analysis of the Chemical Crosslink Structure and Rubber–Filler Interaction Using the Kraus Equation

The fillers in the rubber matrix function as reinforcement agents; reinforcements are caused by generating interaction between the rubber and the filler. Since these interaction led to the immobilization of the rubber chain, it can be difficult to extract the rubber even with a good solvent [36,37]. Therefore, the rubber–filler interaction must be quantitatively calculated using the Kraus equation, which refers to the effect exerted by the swelling of the rubber via the fillers. The Kraus interaction parameter (m) was calculated using linear regression analysis because an increase in the amount of added carbon black resulted in linearly decreased ratios between the volume fraction of the carbon black filler in the NR sample ($\nu_{r}$) and the volume fraction of unfilled carbon black in the NR sample ($\nu_{r-f}$). As a result, calculating the Kraus interaction parameters, which are implied in the rubber–filler interaction, and the total crosslink densities could be divided into the chemical crosslink density and the rubber–filler interaction. In other words, the $\nu_{r-f}$ values obtained from the Kraus equation can be substituted into the Flory–Rehner equation.

**Figure 2:**
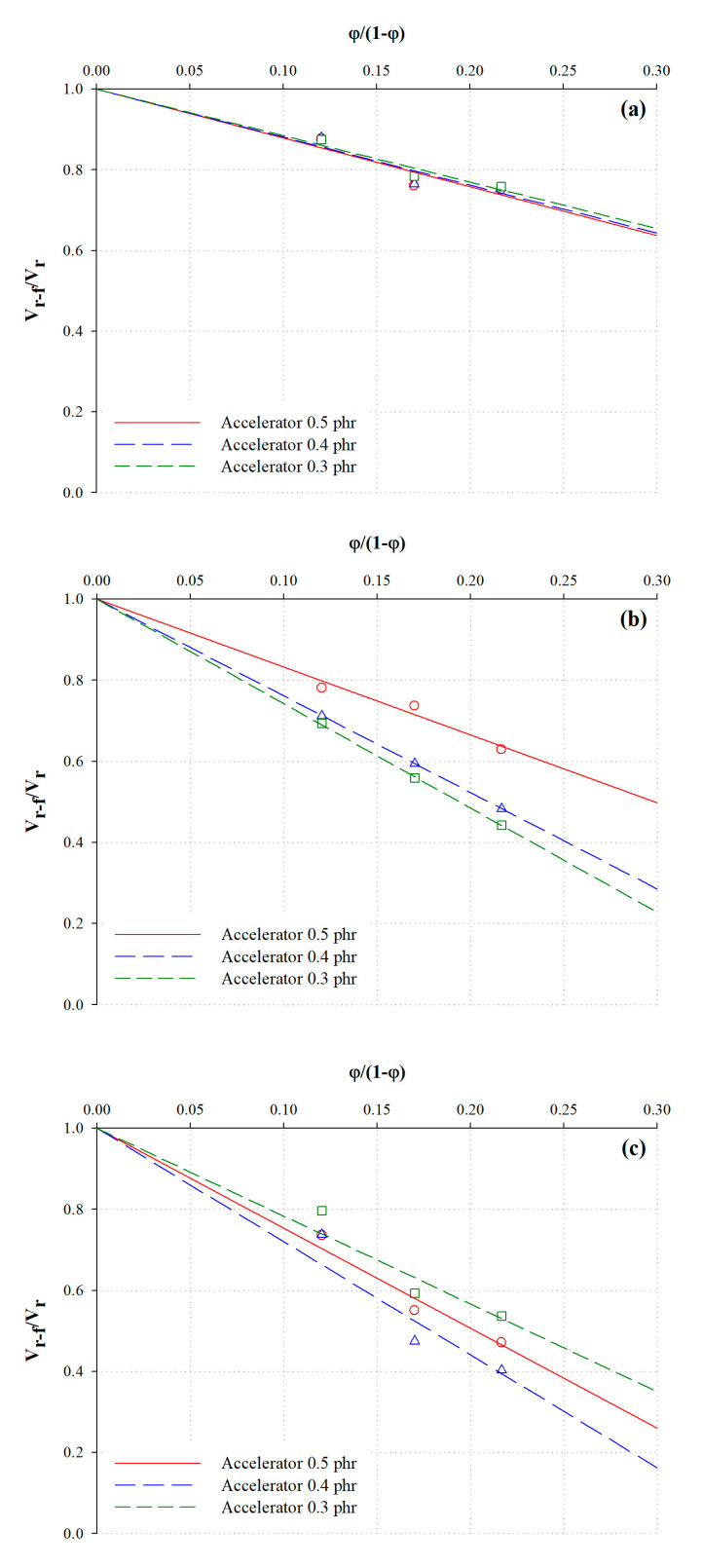
Kraus plot of NR with varying carbon black content (**a**) before sulfide cleavage, (**b**) after polysulfide cleavage, and (**c**) after cleavage of both disulfide and polysulfide.

**Figure 3:**
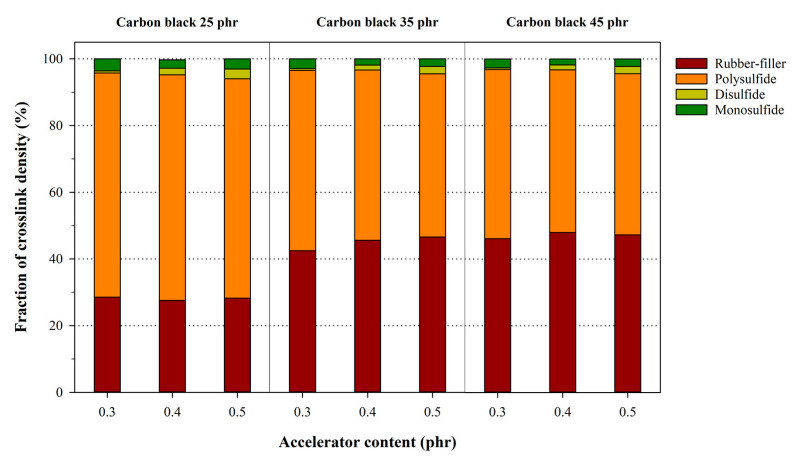
Fraction of the chemical crosslink density and rubber–filler interaction in the NR samples with varying accelerator and carbon black content.

**Figure 4:**
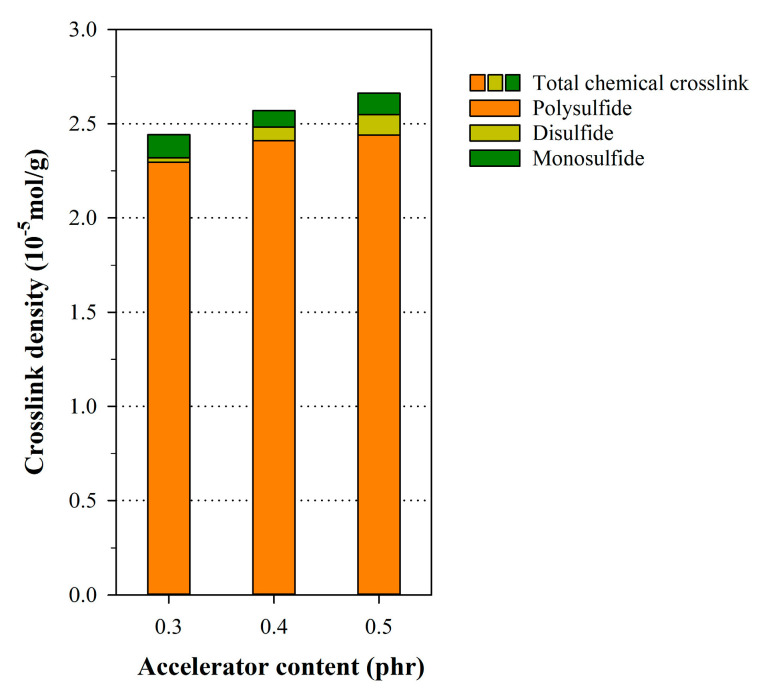
Chemical crosslink structure of the NR sample with varying accelerator content.

The Kraus plots of the NR samples with their corresponding carbon black content are shown in Figure 2, while Table 4 summarizes the Kraus parameters for the NR samples. The coefficient of determination ($R^{2}$) of the Kraus plot resulted in an average value of 0.95, which possessed plot linearity. The crosslink density and structure, without any influence from the carbon black content, were calculated by substituting $\nu_{r-f}$ for $\nu_{r}$ in the Flory–Rehner Equation. Figure 3 shows the fraction of the crosslink density with the carbon black versus the accelerator content, and the calculated results are summarized in Table 5. Here, the effects of the carbon black content increased the total crosslink density, which was attributed to the increase in the rubber–filler interaction for the total crosslinking. However, the fractions of the rubber–filler interaction with the same carbon black content are equivalent, despite increases in the accelerator content because the rubber–filler interaction and chemical crosslinking occurs independently. Except for the influence of the rubber–filler interaction, Figure 4 demonstrates the analysis of the chemically crosslinked structure by the accelerator content. The polysulfide structure in the chemical crosslink structures of NR was most constituted. The crosslink reactions of the rubber generally create mainly polysulfide crosslinks between rubber macromolecules, because the sulfur, which is the most stable form of the eight-membered rings, reacts with rubber molecules through a ring-opening reaction [29,38]. The total chemical crosslink density increased as the MBTS and DPG each increased by 0.1 phr due to more crosslinking reactions between rubber and sulfur, which were the result of generating more radicals. Adding DPG as the secondary accelerator facilitated the formation of more polysulfide and disulfide bonds in the crosslinked structure because the amine group of DPG activated the sulfur group of the primary accelerator. Additionally, adding MBTS as the primary accelerator increased the structure density of all samples because MBTS increased the number of reaction sites with the crosslinking agent [2,29,39].

### 3.4. Influence of Crosslink Structures of NR Compounds on Mechanical Properties

**Figure 5:**
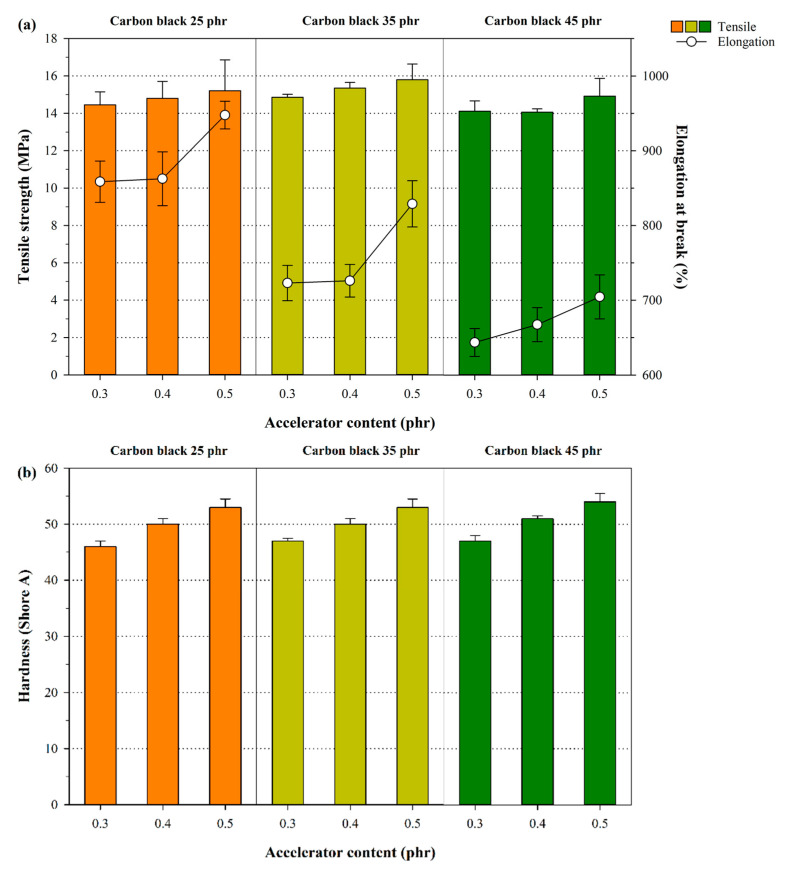
Variation in the mechanical properties in the NR samples by accelerator and carbon black regarding (**a**) tensile strength and elongation at break, and (**b**) hardness.

The mechanical properties of the NR samples were evaluated by focusing on how the crosslink densities and structures were influenced by the accelerator and carbon black content of the rubber. Here, variations in the tensile strength, the elongation at break, and the hardness of the NR samples were noted when the carbon black and accelerator contents in the samples increased (Figure 5). The tensile strength tended to increase to a point when the carbon black content was 35 phr, followed by a notable decrease. This observation was attributed to the reinforcement of the rubber, which was a consequence of the increase in the rubber–filler interaction. After undergoing the initial increase, the subsequent decrease in the tensile strength observed was linked with the reduced dispersion caused by the self-aggregation of carbon black at higher content.

As noted in the results obtained from the chemical crosslink density analysis, the tensile strength slightly increased when the accelerator content was higher. This is due to the formed network structures in rubbers, which arise from the chemical crosslink reaction. A reduced occurrence of elongation at break was observed when the carbon black content was higher. The converse trend was noted at a high crosslinking accelerator content, i.e., the elongation at break was higher when more accelerator was present. This finding was linked with the carbon black content, which reduced the overall softness of the rubber via reinforcement, thereby facilitating the aggregation of carbon black and consequently encouraging the formation of fractures in the rubber. On the other hand, the use of accelerators introduced a higher degree of elasticity into the polymer, which increased the elongation at break effect by increasing the extent of entanglement between the NR chains and the chemical crosslinking. As expected, the hardness of NR increased continuously with the addition of carbon black and the accelerators. In particular, the accelerator has a larger influence on hardness because the chemical crosslinking density of NR was increased by adding the accelerators. In other words, increases in accelerator content cause higher crosslink densities and, therefore, increase the hardness of NR [40].

The influence of crosslink density and structure on the mechanical properties associated with thermal aging was examined by subjecting the NR compounds to the thermal aging process for 70 h at 100 °C. Here, the relative tensile strength and the elongation at break effects were calculated before and after thermal aging. The relative tensile strength and relative elongation at break were calculated before and after aging using the following equation:

(5) $$relative\;tensile\;strength\;\left(\%\right)=\frac{tensile\;strength\;after\;thermal\;aging}{tensile\;strength\;before\;thermal\;aging}\times 100$$

(6) $$relative\;elongation\;at\;break\;\left(\%\right)=\frac{relative\;elongation\;at\;break\;after\;thermal\;aging}{relative\;elongation\;at\;break\;before\;thermal\;aging}\times 100$$

**Figure 6:**
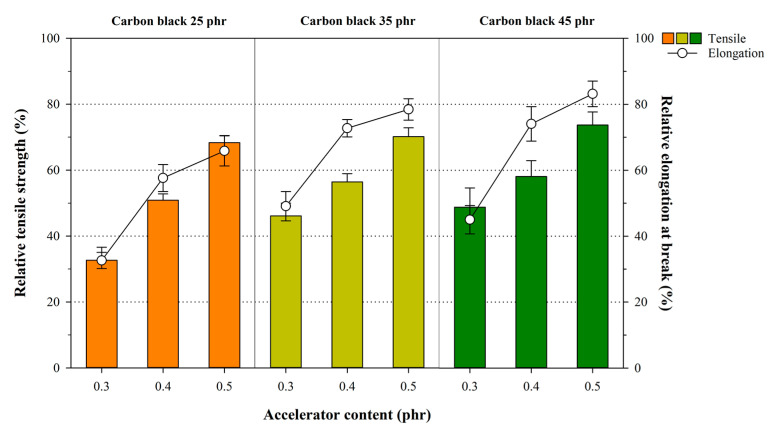
Variation in the relative tensile strength and the elongation at break in the NR samples after thermal aging.

Figure 6 shows the effect of thermal aging on the relative mechanical properties of the crosslink density and structures. Here, the effect of the carbon black in NR compounds on the thermal aging resistance process was insignificant, whereas the accelerator was of significant importance. Most notably, both the relative tensile strength and the elongation at break of the compound sharply increased when DPG was used. In other words, as the content of the accelerator increased, the thermal aging resistance of the material was higher, thereby retaining the initial mechanical properties. This observation was attributed to the dissociation energy of the chemically crosslinked structures, and the formation of new crosslink reactions by the unreacted DPG residues after undergoing thermal aging. In general, the dissociation energy of the chemical crosslink structures by sulfur is the lowest in the polysulfide, followed by the disulfide and the monosulfide. Therefore, the increase in the disulfide and the monosulfide causes a better the thermal resistance in the rubber [38]. In addition, the decrease in the mechanical properties of the material through thermal aging was attributed to the formation of new crosslink reactions by residues such as sulfur and the accelerators after application of the thermal aging process [41,42,43]. However, thermal aging resistance was observed in the sample and became more prominent after adding more accelerator due to the use of thermally stable monosulfides and disulfides as the accelerators [29,44].

## 4. Conclusions

In this study, the crosslink characterization of NR was examined by varying the carbon black and accelerator content of the polymer. Additionally, the correlations between crosslink characterization and the mechanical properties and the effects of thermal aging were studied. To this end, we performed analyses to determine the crosslink density of NR using the equilibrium swelling theory and thiol–amine analysis. The crosslink densities and their associated structures were calculated using the Flory–Rehner and the Kraus equations, and the crosslink characterizations of NR were analyzed by dividing the chemical crosslink density and the rubber–filler interaction. As a result, a distinct correlation was found between the chemical crosslink structures and the mechanical properties, which, in turn, promoted the formation of the crosslink density of the NR rubber. As noted in the results, the tensile strength slightly increased when the accelerator content was higher. In particular, the relative tensile strength and the elongation at break of NR sharply increased by adding the secondary accelerators such as DPG. In other words, the chemical crosslink characterizations of NR were affected by the accelerator content, and these subsequently influenced the rubber’s mechanical properties and thermal aging resistance. In conclusion, the crosslink characterizations of NR can be adjusted by changing the accelerator rather than the carbon black to improve the thermal aging resistance of NR. These results could advance the future development of NR products with optimized mechanical properties, thereby contributing to NR research in general and the fabrication of improved NR products.

## Figures and Tables

**Table 1:** Formulations of plasticized natural rubber (NR) compounds.

Description	NR (wt%)	Carbon Black (phr)	MBTS (phr)	DPG (phr)	Sulfur (phr)	Lubricant (phr)	Antioxidant 1 (phr)	Antioxidant 2 (phr)
NR25-0.3	100	25	0.1	0.2	1.5	1.5	1	2
NR25-0.4	100	25	0.1	0.3	1.5	1.5	1	2
NR25-0.5	100	25	0.2	0.3	1.5	1.5	1	2
NR35-0.3	100	35	0.1	0.2	1.5	1.5	1	2
NR35-0.4	100	35	0.1	0.3	1.5	1.5	1	2
NR35-0.5	100	35	0.2	0.3	1.5	1.5	1	2
NR45-0.3	100	45	0.1	0.2	1.5	1.5	1	2
NR45-0.4	100	45	0.1	0.3	1.5	1.5	1	2
NR45-0.5	100	45	0.2	0.3	1.5	1.5	1	2

**Table 2:** Mixing conditions for the NR compounds.

Mixing Process		Temperature (°C)	Time (min)	Reagents
Kneader	Step 1	-	1	NR
	Step 2	-	3	Carbon black
	Step 3	-	3	Lubricant
	Step 4	90	-	Sulfur
Open-roll	Step 5	30	4	Accelerator

**Table 3:** Curing characteristics of the NR compounds.

Description	t_s2_ (s)	t_90_ (s)	M_H_ (dN·m)	M_L_ (dN·m)	M_H_-M_L_ (dN·m)
NR25-0.3	90	198	20.7	4.7	15.9
NR25-0.4	55	156	22.8	5.5	17.3
NR25-0.5	52	136	22.6	3.1	19.5
NR35-0.3	77	184	22.9	4.6	18.3
NR35-0.4	53	154	24.9	5.1	19.8
NR35-0.5	47	131	25.1	3.7	21.4
NR45-0.3	71	173	25.5	5.5	20.0
NR45-0.4	49	149	26.4	5.2	21.2
NR45-0.5	45	128	28.4	5.0	23.4

**Table 4:** Calculated results of the Kraus plot for NR samples before and after sulfide cleavage.

**Description**	**φ/(1 − φ)**	**Poly + Di + Mono**			**Di + Mono**			**Mono**		
		$\nu_{r}$	$\nu_{r-f}$	m	$\nu_{r}$	$\nu_{r-f}$	m	$\nu_{r}$	$\nu_{r-f}$	m
NR25-0.3	0.1202	0.1655	0.1452	1.2081	0.0540	0.0422	1.6722	0.0395	0.0291	2.4648
NR35-0.3	0.1699	0.1907			0.0572			0.0528		
NR45-0.3	0.2166	0.1932			0.0670			0.0616		
NR25-0.4	0.1203	0.1624	0.1429	1.1873	0.0496	0.0353	2.3837	0.0347	0.0256	2.7934
NR35-0.4	0.1700	0.1867			0.0594			0.0539		
NR45-0.4	0.2168	0.1890			0.0731			0.0635		
NR25-0.5	0.1204	0.1596	0.1396	1.1492	0.0494	0.0335	2.5756	0.0383	0.0305	2.1666
NR35-0.5	0.1702	0.1690			0.0570			0.0514		
NR45-0.5	0.2169	0.1884			0.0775			0.0568		

**Table 5:** Calculated density of the chemical crosslink and rubber–filler interaction in the NR samples with varying accelerator and carbon black content.

Description	Crosslink Density (10^−5^ mol/g)			Fraction (%)	
	Interaction	Chemical	Total	Interaction	Cehmical
NR25-0.3	0.9757	2.4417	3.4174	71.45	28.55
NR35-0.3	1.8023		4.2440	57.53	42.47
NR45-0.3	2.0933		4.5350	53.84	46.16
NR35-0.4	0.9927	2.5705	3.5632	72.14	27.86
NR45-0.4	2.1555		4.7260	54.39	45.61
NR45-0.4	2.3713		4.9418	52.02	47.98
NR25-0.5	1.0491	2.6626	3.7117	71.74	28.26
NR35-0.5	2.3245		4.9871	53.39	46.61
NR45-0.5	2.3852		5.0478	52.75	47.25
